# Clinically relevant topography of the great saphenous vein and saphenofemoral junction, a study from adult embalmed cadavers

**DOI:** 10.12688/f1000research.178143.1

**Published:** 2026-03-04

**Authors:** Latha V Prabhu, B.V. Murlimanju, M. Praveen Shenoy, Mangala M Pai, Mamatha Tonse, Ashwin R Rai

**Affiliations:** 1 Department of Anatomy, Kasturba Medical College Mangalore, Manipal Academy of Higher Education, Manipal, India

**Keywords:** Great saphenous vein, Saphenous opening, Saphenous nerve, Saphenofemoral junction, Venous cut down

## Abstract

**Background:**

Knowledge of the morphology of the great saphenous vein (GSV) is important because of its clinical applications and involvement in venous diseases. In this study, the aim was to determine the morphology and topography of the saphenous opening and to perform side-based and gender-based comparisons. The objective of this study was to describe the precise topography of the GSV with respect to the medial malleolus and saphenous nerves.

**Methods:**

This is an institution-based cross-sectional study including 40 lower extremities from 20 adult embalmed cadavers. The morphometric data was obtained by applying a digital Vernier caliper.

**Results:**

The shape of the saphenous opening was noted. In 29 extremities (72.5%), the saphenous opening was vertically oval, with a round shape in 8 (20%) and a kidney shape in 3 (7.5%). There was no statistically significant difference (p>0.05) between the right- and left-sided morphometric data. The present study observed that females had smaller dimensions of the saphenous opening and it was more supero-medially placed than in males (p<0.05) in relation to the pubic tubercle. In 24 lower extremities (60%), the saphenous nerve ran anterior to the GSV, and in the remaining 16 (40%), the saphenous nerve was divided into two branches running anterior and posterior to the GSV between the knee and ankle joints.

**Conclusion:**

The present study provides important data on the morphology and topography of the saphenous opening and GSV in relation to the pubic tubercle, medial malleolus, and saphenous nerve. However, the data would be more accurate with a larger sample size.

## Introduction

The great saphenous vein (GSV) drains into the femoral vein at the saphenofemoral junction (SFJ). The valves in the SFJ maintain the unidirectional flow of blood, preventing backflow and venous insufficiency. ^1– 3^ Knowledge of SFJ's anatomy of the SFJ is crucial for treating venous diseases and successful postoperative outcomes. ^4, 5^ It has been described that the SFJ can have significant anatomical variations, the prior knowledge of which is crucial to prevent iatrogenic injuries and to plan the surgery. Radiological investigations have revealed that the SFJ region is more complex than that previously described. Hence, accurate identification of the detailed anatomy and knowledge of anatomical variations are necessary while addressing varicose veins. ^6^ It has been reported that inadequate identification of the topography of the GSV and misinterpretation of the SFJ can lead to varicose veins. ^7, 8^

The distal part of the GSV is often utilized for venous access during procedures such as percutaneous cannulation or venous cutdown and strip of varicosities, where iatrogenic injury to the saphenous nerve should not occur. A literature review revealed that studies regarding the morphology of the GSV are scarce, particularly from our sample population with respect to its relation to the saphenous nerve (SN) and SFJ. The morphology and topography of the GSV have clinical implications, including accurate canulation, involvement of the saphenous nerve during stripping surgery, involvement of the GSV and SN in ulcer formation at the medial malleolus and on the dorsum of the foot, and ligation of perforators at the ankle, which summons more anatomical studies in this region.

These are the rationale for performing this anatomical research, and the goal of this anatomical study was to record the morphology and topography of the saphenous opening and to perform side- and gender-based comparisons of the variability. The objective of this study was to investigate the topography of the distal GSV with respect to the medial malleolus and the SN.

## Methods

This institutionally based cross-sectional anatomical study performed between 2024 and 2025 included 40 adult lower extremities from 20 adult embalmed cadavers. Of these, ten were male and female cadavers. The ethnicity of the population studied belonged to Dravidian descent. Only adult embalmed cadavers from the South Indian population were included, and the lower extremities with any obvious visible pathology were excluded. A convenient sampling method was considered, that is, the number of specimens available in our department. The Institutional Ethics Committee of Kasturba Medical College, Mangalore, India Reg. No. ECR/541/IND/KA/2014/RR-20 (IEC KMC MLR 09/2024/568, dated 19/09/2024) was approved and permitted for this study. The cadavers utilized in this study belonged to the department of anatomy of our institution. These adult cadavers were from donated bodies and the written informed consent for the utilization of them for the purpose of medical teaching and medical research was obtained during the time of donating the body. The protocol of this study was archived in dx.doi.org/10.17504/protocols.io.j8nlk16qxg5r/v1.

**Figure 1.**
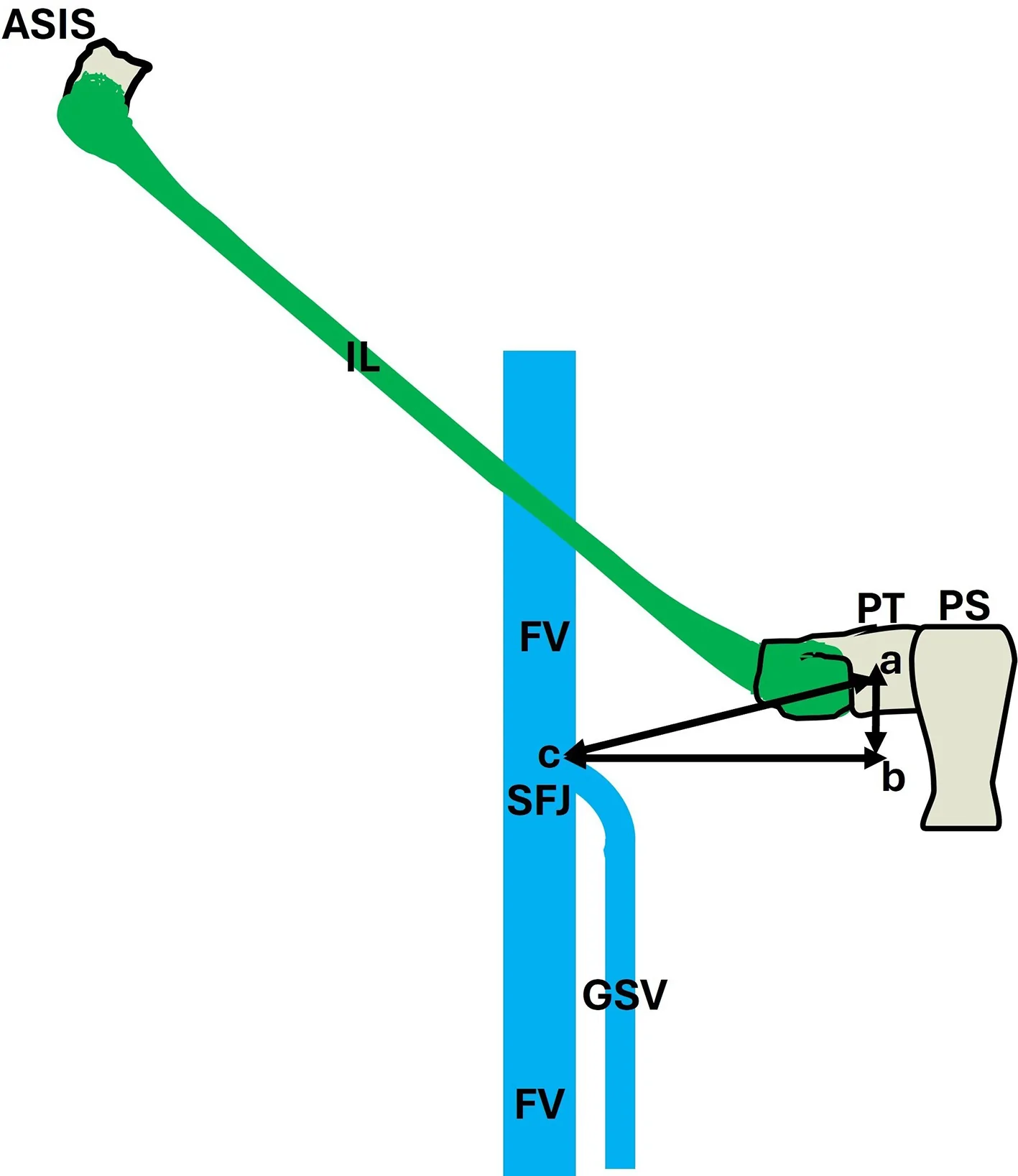
Schematic diagram showing the topographic details of the saphenofemoral junction (SFJ), which are collected in this study; ab-vertical distance; bc-lateral distance; ac-oblique distance of SFJ from the pubic tubercle (PT); ASIS-anterior superior iliac spine; IL-inguinal ligament; PS-pubic symphysis; GSV-great saphenous vein; FV-femoral vein.

**Figure 2.**
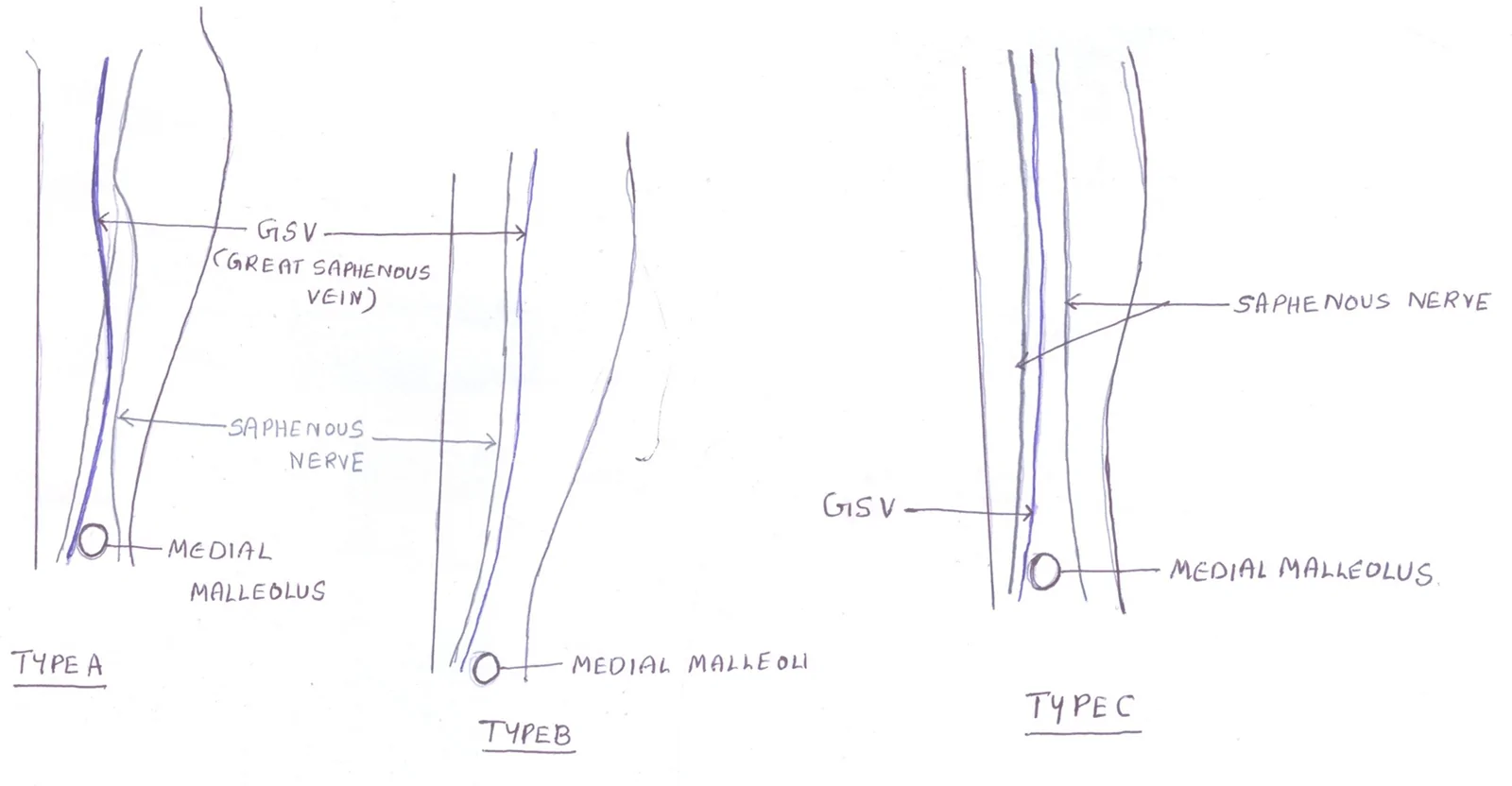
Schematic representation of the relation of GSV and saphenous nerve as per Wilmot and Evans classification.

Different shapes of the saphenous openings were identified. A digital vernier caliper (Mitutoyo, Japan) was used to perform the measurements in this study, which included the vertical length and width of the saphenous opening, and the vertical (ab in Figure 1), lateral (bc in Figure 1), and oblique (ac in Figure 1) distances of the SFJ from the pubic tubercle. A vertical plane was drawn from the saphenous opening, and a horizontal plane was drawn from the pubic tubercle. The meeting point of these two lines was used to measure the vertical and lateral distances of the SFJ from the pubic tubercle. The horizontal distance between the GSV and the midpoint of the medial malleolus was determined. The diameter of the GSV was then measured. The relationship between the saphenous nerve and GSV was studied based on the classification by Wilmot and Evans, ^5^ which is shown in Figure 2.

Only one author performed all measurements to prevent inter-observer errors. Three measurements were recorded for each dimension, and their average was considered the final measurement to prevent intra-observer bias. SPSS version 29 (IBM, USA) was used for statistical analysis. The paired t-test and independent sample t-test were used for comparisons ( Tables 1 and 2).

**Table 1.** Side-based comparison of the morphometric and topographic data of the saphenofemoral junction (SFJ).

Parameter measured	Right side	Left side
Vertical length of saphenous opening	3±0.4 cm	2.7±0.4 cm
Width of saphenous opening	1.7±0.5 cm	1.5±0.5 cm
Lateral distance of SFJ from pubic tubercle	3.8±0.9 cm	3.6±1.3 cm
Vertical distance of SFJ from pubic tubercle	1.8±0.8 cm	2.4±1 cm
Oblique distance of SFJ from pubic tubercle	4.3±0.7 cm	4.3±1.4 cm

Statistical analysis using paired samples t-test; no statistical significance (p>0.05).

**Table 2.** Gender-based comparison of the morphometric and topographic data of the saphenofemoral junction (SFJ).

Parameter measured	Male	Female
Vertical length of saphenous opening	1.7±0.8 cm	1.6±0.1 cm
Width of saphenous opening *	0.9±0.4 cm	0.5±0 cm
Lateral distance of SFJ from pubic tubercle *	3.7±0.9 cm	3.3±1.3 cm
Vertical distance of SFJ from pubic tubercle *	2±0.4 cm	1.4±0.7 cm
Oblique distance of SFJ from pubic tubercle *	4.1±0.6 cm	3.6±0.9 cm

Statistical analysis-independent samples t-test;

* statistically significant difference exists (p<0.05).

## Results

**Figure 3.**
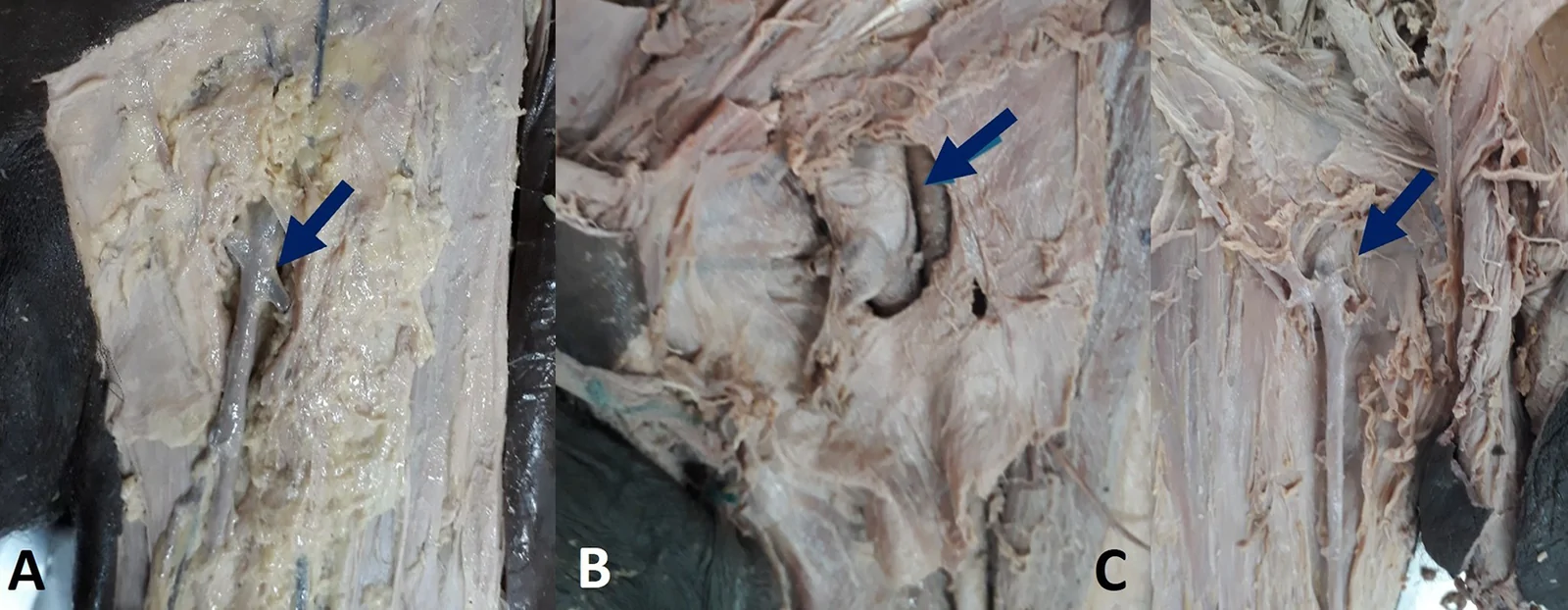
Lower limbs of the embalmed cadavers showing the vertically oval shaped saphenous opening (A); round shaped saphenous opening (B) and the kidney shaped saphenous opening (C).

In 29 extremities (72.5%), the saphenous opening was vertically oval ( Figure 3A), round in 8 (20%, Figure 3B), and kidney in 3 (7.5%, Figure 3C). A side-based comparison of the measured parameters is presented in Table 1, and there was no significant difference (p > 0.05). Table 2 presents a sex-based comparison of the measurements. In the present study, females had small saphenous opening dimensions (p < 0.05), and the saphenous opening was more supero-medially placed in females than in males (p < 0.05).

**Figure 4.**
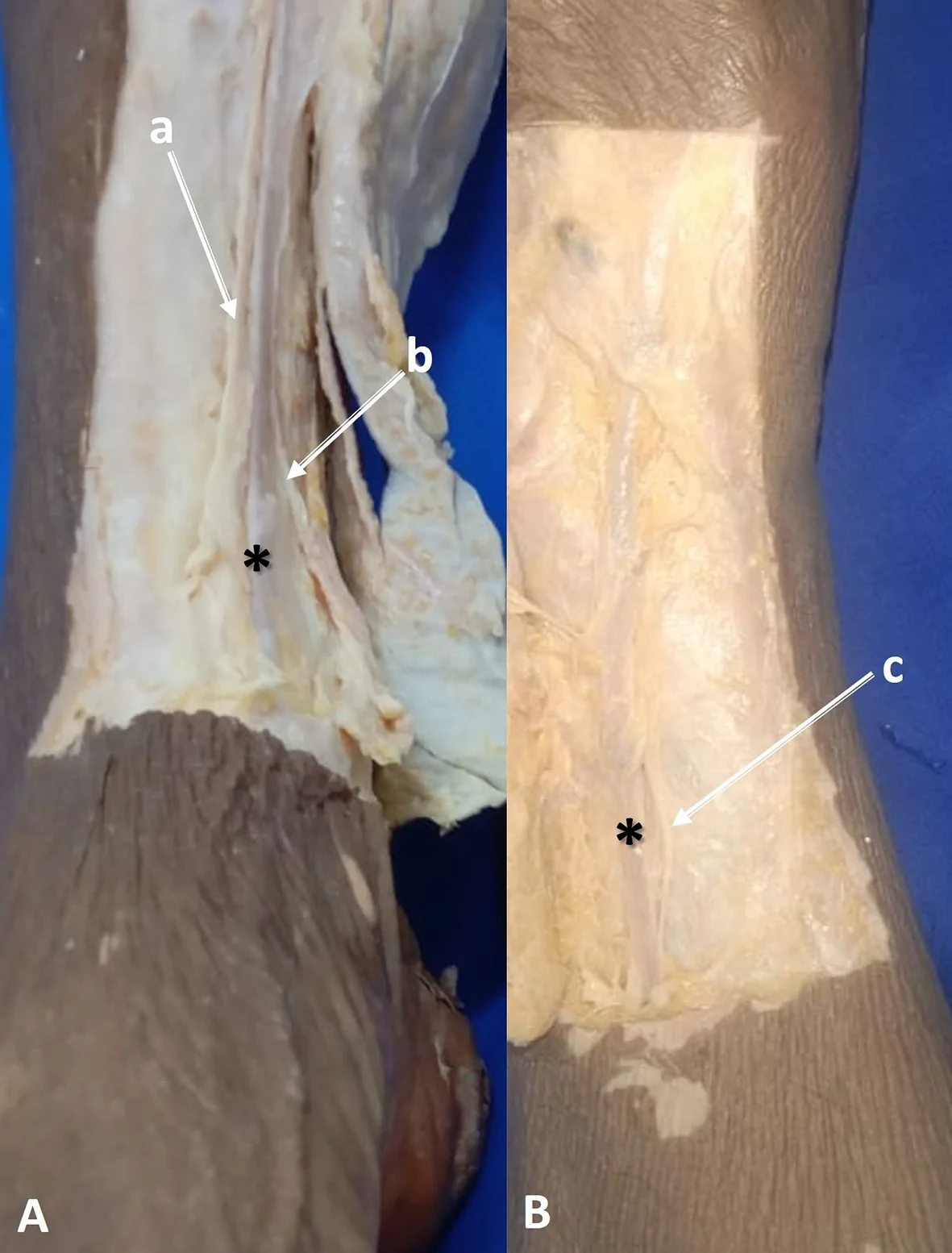
A. A branch of saphenous nerve running anterior to GSV and another branch of it running posterior (type A), observed in 16 lower limbs (40%); B. Saphenous nerve was running anterior to GSV (type B), observed in 24 lower limbs (60%).

In 24 lower extremities (60%), the saphenous nerve ran anteriorly to the GSV (type B, Figure 4B), and in the remaining 16 (40%), the saphenous nerve was divided into two branches (type A, Figure 4A) running anteriorly and posteriorly to the GSV between the knee and ankle joints. Type C pattern, as per the Wilmot and Evans classification, ^5^ was not observed in this study. The distance between GSV and medial malleolus was 2.34 ± 0.75 cm on the right side and 2.29 ± 0.52 cm on the left side. The depth of GSV from the skin was 0.31 ± 0.07 cm and 0.28 ± 0.09 cm on the right and left lower limbs. The diameter of the GSV near the medial malleolus measured 0.41 ± 0.09 cm on the right lower extremity and 0.38 ± 0.05 cm on the left lower extremity.

## Discussion

The GSV pierces the cribriform fascia at the lower corner of the falciform margin of the saphenous opening and terminates in the femoral vein. The saphenous opening is a defect in the fascia lata of the thigh in the upper part of the femoral triangle. The usual type of saphenous opening is described as ‘vein star’ shape, however as per the study by Ndiaye et al. ^9^ this shape was present in only 10% cases. However, the literature review did not reveal studies on the different shapes of the saphenous opening. The present study can be considered novel from this perspective, as three different shapes are observed. Vertically oval, round, and kidney-shaped saphenous openings are reported in this study. This knowledge will add to the existing literature as the saphenous opening is a complex structure with significant morphological variability, which is important for clinical procedures involving the GSV. GSV is considered as the longest vein in the human body, which is formed by the joining of the medial marginal vein and the medial end of the dorsal venous arch of the foot, runs superiorly just anterior to the medial malleolus, followed by the medial aspects of the leg and thigh. ^10, 11^ In an ultrasound observation, it was reported that the center of the SFJ was found to be at 1 ± 0.9 cm inferiorly and 2.4 ± 0.6 cm laterally from the pubic tubercle. ^5^ However, it has been reported that this location can vary slightly based on factors such as sex and body composition. The junction was slightly proximal to the pubic tubercle in females in comparison to males. ^5^ In this anatomical research, it was observed that the SFJ was more supermedially placed in females than in males ( Table 2). The present study reports both side- and gender-based data on the topography of the saphenofemoral junction. Statistical significance was determined by comparing the data for the right and left, lower limbs ( Table 1). The SFJ can have one to ten tributaries, with a median number of four. ^7, 8^ These include the superficial and deep external pudendal veins, superficial epigastric vein, and superficial circumflex iliac vein. ^8^ The GSV can be bifid in approximately 18.1% of cases, meaning it splits into two trunks at the SFJ. ^7^ There are few reports that suggest that the external pudendal artery runs in front of the GSV. In the present study, this anatomical variation was not observed, which may be due to the smaller number of samples being studied.

The complicated anatomy and morphological variations at the SFJ can lead to significant challenges during surgery, such as the risk of missing tributaries or causing iatrogenic trauma to the surrounding structures. Failure to identify the tributaries and their ligation can lead to recurrence of varicose veins. ^8, 12^ Detailed anatomical knowledge allows for better preoperative evaluations, ensuring that patients with anatomical variations receive accurate surgical procedures. ^13^ Preoperative ultrasound and CT venography can help detect the venous anatomy and its anatomical variations, such as the unusual location of the GSV. ^14, 15^

Venous cutdown was performed to gain access to the GSV. There are different types of vascular access, such as percutaneous, ultrasound-guided, and intraosseous. Lack of insight of the surface anatomy and dimensions of GSV can cause difficulty in these procedures and may demand more time consumption for GSV access. ^16^ It was described that the distal great saphenous vein runs 2.5 cm in front of the medial malleolus, 4 mm deeper to skin and presents a diameter of 4 mm. ^17^ The GSV was 2.34 ± 0.75 cm and 2.29 ± 0.52 cm anterior to the medial malleolus over the right and left sides in the present study. The depth of GSV from the skin was 0.31 ± 0.07 cm and 0.28 ± 0.09 cm over the right and left sides. The diameter near the medial malleolus was 0.41 ± 0.09 cm on the right and 0.38 ± 0.05 cm on the left lower limbs of this study. The limitations of these data include embalming, which may have altered their dimensions. However, the data are comparable to the previous clinical study of saphenous venous grafts for cardiothoracic surgery, where it was 0.42 cm in diameter. ^18^ In the present study, 60% of lower extremities had the saphenous nerve running anterior to the GSV which is type ‘B’ of Wilmot and Evans classification and in the remaining 40%, the saphenous nerve divided into two branches (type A) and running anterior and posterior to GSV, between the knee and ankle joints. The type C pattern of the Wilmot and Evans classification, ^5^ where branching of the saphenous nerve occurs in the thigh region, was not observed in the present anatomical study.

Sensory disturbances in the saphenous nerve distribution after the stripping procedure of GSV have been reported in clinical literature. In clinical research, the GSV was stripped upward in one leg and downwards in the other, and the comparison was performed. It was finally opined that the stripping of GSV upwards could lead to a significant sensory deficit than inferiorly. ^19^ It is overall suggested that stripping of the distal part of the GSV could be avoided to reduce the risk of damage to the saphenous nerve. ^20^ In this context, the morphological and topographic data obtained from this study may be of clinical importance and can assist the operating surgeon with better outcomes. The data may be considered a morphological database of our sample population. However, the present study has certain limitations like the samples are formalin fixed cadavers, which might have alteration in the dimension because of the embalming. Another limitation of this study is the small sample size, and the data may be more accurate with a larger sample size.

## Conclusion

A detailed understanding of SFJ morphology and topography is vital for clinicians to perform successful interventions and effectively manage venous disorders. It is essential to understand the relationship between the GSV and the saphenous nerve, GSV, and bony landmarks, such as the medial malleolus. In this context, anatomical details obtained from this study can be useful in procedures such as stripping surgeries of the GSV, thermal ablation of varicosities, venesection, and canalization in acute emergencies.

## Ethical statement

The authors state that every effort was made to follow the institutional and international ethical guidelines and laws pertaining to medical research.

## Data Availability

Underlying data repository name: [Morphometry of saphenofemoral junction] https://doi.org/10.6084/m9.figshare.31306825. ^21^ The project contains the following underlying data: [Saphenous vein topography] (Raw Data). Data is available under the terms of the Creative Commons By 4.0 License (CC-BY 4.0).
