# Supplementary material for: Clinically relevant topography of the great saphenous vein and saphenofemoral junction, a study from adult embalmed cadavers
Source: F1000Res. 2026 Mar 4;15:349. [Version 1] doi: 10.12688/f1000research.178143.1 (PMC13555047; doi:10.12688/f1000research.178143.1)
Supplement: Peer review history

# Reviewer report: Reviewer response for version 1

**DOI:** 10.5256/f1000research.196496.r466796

**Published:** 2026-04-01

**Authors:** Abdalla Ahmed Eldaw Elamin

**Affiliations:** 1 Ras Al Khaimah Medical and Health Sciences University College of Medical Sciences (Ringgold ID: 286652), Ras Al-Khaimah, United Arab Emirates

This study highlights anatomical variations of the saphenous opening and the relationship of the saphenous nerve to the great saphenous vein. The authors measure the width and length of the saphenous opening, describe its range of shapes, and determine the distance of the saphenofemoral junction from the pubic tubercle. They also assess the diameter of the great saphenous vein near the medial malleolus and measure its distance from the medial malleolus. In addition, the study presents a bilateral and gender-based comparison of the saphenous opening. Overall, it provides a useful reference for dissection, imaging, and preoperative planning.

 The second paragraph of the introduction is not supported by any references, and the overall introduction appears brief. Figure 1 is included in the introduction; however, it would be more appropriate to place it in the Methods section. The quality of Figure 2 is low and should be improved.

 For better clarity, it is recommended to add clear demarcations between A, B, and C in Figure 3 and to change the arrow color in Figure 4. Additionally, in figure 3, outlining the margin of the saphenous opening with color or dashed lines would improve clarity. The repeated content related to limitations in the discussion section should be removed. Furthermore, the discussion does not address the results of the bilateral and gender comparisons; these findings should be contrasted with previous studies. Kindly provide the complete bibliographic details for the last reference.

Is the work clearly and accurately presented and does it cite the current literature?

Partly

If applicable, is the statistical analysis and its interpretation appropriate?

Yes

Are all the source data underlying the results available to ensure full reproducibility?

Yes

Is the study design appropriate and is the work technically sound?

Yes

Are the conclusions drawn adequately supported by the results?

Yes

Are sufficient details of methods and analysis provided to allow replication by others?

Yes

Reviewer Expertise:

Stereology, Anatomy, Electromagnetic field effects on organsims, Peripheral nerve regeneration.

I confirm that I have read this submission and believe that I have an appropriate level of expertise to confirm that it is of an acceptable scientific standard, however I have significant reservations, as outlined above.

# Response

**Published:** 2026-05-11

**Authors:** Mamatha Tonse

**Affiliations:** Anatomy, Kasturba Medical College Mangalore, Mangaluru, Karnataka, India

Dear Sir,

 Thank you so much for the peer review of our manuscript and the expert comments. The manuscript has been revised as per your suggestion.

 In the methodology section, the identification of the great saphenous vein in the saphenous fascia and dissection within the saphenous compartment, between the superficial fascia and the muscular fascia was added as per the reviewer’s opinion. The veins located superficial to the saphenous fascia were not considered as the great saphenous vein. It was also mentioned in the methodology that the present study does not report on the presence of accessory anterior saphenous veins or the Giacomini vein. The study limitation is added in the discussion section regarding the widespread availability of high-resolution ultrasonography and other imaging techniques for the more precise and clinically relevant data in living subjects. Few more recent references were cited for the introduction section. The Figure 2 was rewritten and Figures 3-4 were revised as per the suggestions of the reviewer. The discussion was revised with more description on the side-based and gender-based variations. The limitations were revised. The last reference (of the dataset) was given in full.

# Reviewer report: Reviewer response for version 1

**DOI:** 10.5256/f1000research.196496.r464718

**Published:** 2026-03-11

**Authors:** Marian Simka

**Affiliations:** 1 University of Opole, Opole, Poland

This manuscript presents a study on the anatomy of the great saphenous vein based on dissections of cadaveric specimens. While such investigations were commonly performed in previous centuries, the relevance of this methodology is questionable in the context of modern anatomical and clinical research. With the widespread availability of high-resolution ultrasonography and other imaging techniques, it is now possible to obtain more precise and clinically relevant data in living subjects. Consequently, it is unclear whether the present cadaver-based approach can provide novel or meaningful scientific insights.

 Another major concern relates to the identification of the great saphenous vein. According to the current anatomical definition, the great saphenous vein is located within the saphenous compartment, between the superficial (saphenous) fascia and the muscular fascia. Veins located superficial to the saphenous fascia should not be considered the great saphenous vein. The authors do not specify whether the saphenous fascia was identified during dissection. This structure is extremely difficult to reliably identify in embalmed cadavers, which is one of the reasons why such anatomical studies are rarely performed today, as more accurate investigative methods are available. Without clear confirmation that the saphenous compartment was identified, it remains uncertain whether the examined vessels were indeed the great saphenous vein rather than adjacent superficial veins.

 Furthermore, the manuscript does not report the presence of accessory anterior saphenous veins or the Giacomini vein. Although these anatomical structures are not present in all individuals, they are relatively common and would be expected to appear in at least some specimens. Their absence from the description raises additional concerns regarding the accuracy and completeness of the dissections.

Is the work clearly and accurately presented and does it cite the current literature?

No

If applicable, is the statistical analysis and its interpretation appropriate?

Not applicable

Are all the source data underlying the results available to ensure full reproducibility?

No source data required

Is the study design appropriate and is the work technically sound?

No

Are the conclusions drawn adequately supported by the results?

No

Are sufficient details of methods and analysis provided to allow replication by others?

Partly

Reviewer Expertise:

Dr. Marian Simka, specialist in angiology and general surgery, is the associate professor of the Department of Anatomy at the University of Opole, Poland and the associate professor of the Department of Nursing at the College of Applied Sciences in Ruda Śląska, Poland. He is the author of over 200 publications in the field of anatomy, angiology, phlebology, wound healing, vascular surgery and interventional radiology. His research focuses at the pathophysiology, diagnostics and treatment of venous pathologies.

I confirm that I have read this submission and believe that I have an appropriate level of expertise to state that I do not consider it to be of an acceptable scientific standard, for reasons outlined above.
